# Health and Disease in Relation to Marriage and the Married State

**Published:** 1906-09

**Authors:** 


**Health and Disease in Relation to Marriage and the Married State.** Edited by Dr. H. Senator and Dr. S. Kaminer. Translated by J. Dulberg, M.D. Vol. II. Pp. 775. New York and London: Rebman Limited. [1905.]

The second volume of this work has now been issued, and lies before us. Like the first volume, it consists of a number of monographs by different authors.

It opens with two papers respectively by Dr. A. Neisser and Dr. R. Ledermann on gonorrhœal diseases and syphilis, and the effects these contagious diseases may have on the male and female organs with regard to the marriage of either party. Questions of interest on both subjects are fully discussed, especially that of sterility in marriage arising from these causes. A most important point Dr. Neisser considers is that the lay public is still disinclined to recognise the importance of gonorrhœa and the necessity of treating it seriously from the first. The question naturally arises in our minds that this may be so, but how are we to educate the public to a due appreciation of the fact with the feeling there is in this country on these subjects?

It has been proposed in Germany that the law should imprison for not less than two years and deprive of their civil rights anyone knowingly suffering from contagious sexual disease having connection with a woman. There seems much to be said, perhaps, on this point. When, however, "the man in the street," who considers himself perfectly competent to give judgment in the most intricate cases of theology and morals, who is the organiser of Purity Societies, Vigilance Committees, &c., did all he could to repeal the C.D. Acts, there does not seem much chance of legal restraint of the mildest kind being obtained against this crying scandal.

Professor A. Hoffa follows on "The Diseases of Locomotion," pelvic deformities, of course, being amply referred to, the paper being one of importance. Professor C. Posner's paper on "Diseases of the Lower Genital Organs, including Physical Impotence," discusses several obscure questions. Dr. L. Blumreich, in "Diseases of Women, including Sterility," gives a very useful monograph discussing fully the diseases of these parts. Dr. Eulenburg, in his paper on "Diseases of the Nervous System," treats of those strange nervous conditions found in women, so often complicated with hysteria, and which constantly come before the general practitioner, giving him much anxiety and trouble. Dr. E. Mendel treats of "Insanity in Relation to Marriage." He has collected some interesting statistics. Little is added to the knowledge that specialists possess in this country and to the conclusions they have arrived at as to the marriage of persons who have either themselves been insane or in whose families there is insane heredity or tendency thereto.

Dr. Moll's paper on "Perverse Sexual Sensations and Psychical Impotence" is, to a large extent, a *résumé* of his well-known works on the subject. Practitioners in the past considered these subjects as best left on one side, or, if considered, only to be treated in the privacy of the study. Patients consulting them were treated as if their complaints were of no moment or entirely fanciful. Little or nothing was understood of the deep psychical complexity of their feelings, and futile advice, or even worse, was given. That day is gone by, and however we may feel inclined to fight shy of the subject, the alienist and the neurologist finds he must face them. On the Continent for many years past the matter has been considered plainly and scientifically, being treated of as one very serious to the patient in many ways. Its bearing on the question of marriage is well set forth in this paper.

Dr. S. Placzek's paper on "Medico-Professional Secrecy in Relation to Marriage," from the ethical standpoint, shows the difference of the law in Germany and in England.

Leibnitz said, "Marriage is a good thing, but a wise man ought to consider of it all his life." The papers in these two volumes seem to go some way to support the second half of the proposition.

